# Addressing psychiatric care in conflict zones: recommendations for the Arab region

**DOI:** 10.1192/bji.2020.41

**Published:** 2021-02

**Authors:** Joseph El-Khoury, Andres Barkil-Oteo, Lynn Adam

**Affiliations:** Department of Psychiatry, American University of Beirut, Beirut, Lebanon. Email: je47@aub.edu.lb

**Keywords:** Arab countries, Middle East, conflict, mental healthcare, psychiatric care, international organisations

## Abstract

The Arab world has struggled with conflict and political turmoil for several decades, rendering its already underdeveloped mental healthcare system unable to serve the psychiatric needs of victims of violence and trauma, with consequences that extend far beyond the cessation of hostilities. This role has become incumbent on international relief agencies, which have expanded mental health programmes in countries of conflict and refuge. Although their intervention has overall been positive, their mission is usually short term, leaving countries unable to maintain these advantages when the funding ends. The authors advocate for a sustainable framework that emphasises a larger role for regional and local actors. Expertise that is culturally and socially grounded could take the initiative in research, training and deployment in collaboration with non-governmental organisations, allowing for comprehensive development of the mental health sector.

## Background

The Middle East is an area that has been marred by waves of armed conflict for most of the 20th century, through the fall of the Ottoman empire, the era of decolonisation, the Arab–Israeli conflict and the various internal and external wars that are ongoing. It is also a region where mental ill health is heavily stigmatised and psychiatric care is underdeveloped.^1^ Whereas healthcare systems have undergone a radical shift in the western hemisphere and other developed economies, psychiatrists in the majority of Arab countries provide care out of private clinics to a privileged few, while severe mental disorders are treated in large asylum-style institutions.^2^ This has meant that insufficient attention has been given to the evolving needs of the local population and the context of care provision.

At the time of writing, eight (Yemen, Sudan, Somalia, Saudi Arabia, Iraq, Syria, Palestinian territories, and Libya) out of 20 states of the Arab league of nations are involved in internal or transnational armed conflicts directly or indirectly affecting their civilian populations. This figure disregards political instability and social unrest, and countries that have witnessed intermittent terrorist activities on their territory in the past few years. Apart from Saudi Arabia, these conflict-ridden countries happen to be some the most under-resourced in terms of specialist workforce and facilities.^3^

## The psychiatric burden of conflict

In view of this situation, it is not surprising to find a lack of proper estimation or understanding of the psychiatric burden resulting from these conflicts. A systematic review of 157 articles revealed a greater focus on domestic violence than on other forms of trauma.^4^ Another review found that post-traumatic stress disorder (PTSD) and major depression were the most common presentations, while acknowledging the limited population samples on which these studies were based.^5^

Victims of conflict can be categorised into direct victims of violence, witnesses of violence, refugees, and relatives of the deceased and injured. Psychiatrists and policy makers have traditionally relied on international classification systems for diagnosis and treatment decisions. The DSM is now on its fifth edition, and the 11th version of the ICD was released in 2018. The past decade has witnessed an emphasis on strengthening the validity of these systems by including databases and expertise from developing countries, including the Arab region.^6^ Yet, the framing of stress-related disorders in a cultural context remains challenging. Rates of PTSD in Syrian refugees range from 30% to 60% on standardised assessments, which is not accurately reflected in clinical presentations or in treatment-seeking behaviour.^7–9^ Some argue for the prominence of the new category of complex PTSD in this population.^10^ Clinical reports^11^ and research confirm that the classic PTSD formulation has many limitations in capturing the symptoms of psychological trauma in refugees.^12^ van der Kolk and colleagues have demonstrated that the variety of clinical manifestations of people with ‘complex trauma’ histories can be missed when adhering to the official PTSD classification, whether using the ICD or the DSM.^13^ In fact, ‘pure’ PTSD with prominent intrusive thoughts and avoidance symptoms is ‘the exception rather than the rule’.^14^ This is crucial in the context of refugees who are subjected to ongoing traumas following the initial index event, if any, and may not achieve a sense of safety for years. Similar to victims of natural disasters, the prognosis of classic PTSD has as much to do with events that occur after the trauma as with the trauma itself.^15^

Delay in seeking diagnosis and treatment through formal medical assessments is already common in Arab countries. In situations of instability, such as forced migration or constant military threat and the dearth of psychiatric care, coupled with the prioritisation of other medical and social needs, are likely to mean that many cases remain undetected. The most severe presentations where intervention is unavoidable will rely on self-treatment or suboptimal primary care consultations. As a consequence, treatable psychiatric disorders can convert into chronic conditions, affecting individual quality of life and family members well beyond the duration of the conflict. Deterioration in physical health and risk of substance misuse through unmonitored dependence on tranquilisers are often consequences of this neglect.

## The role of global agencies

The international response generated by the Iraqi and Syrian conflicts led to the involvement of relief agencies in supporting the mental health of millions of internally displaced and trans-border refugees in neighbouring countries such as Turkey, Jordan and Lebanon, but also on European shores. It also brought a renewed interest in researching psychological trauma in these populations.

In already fragile health economies, the onset of political instability or warfare, including that in a neighbouring country, can stretch services to all but the point of collapse. In the resulting vacuum, local and international non-governmental organisations (NGOs) have increased their footprint and have often become the primary provider of mental health services, including routine psychiatric care. These organisations have added mental healthcare to their core services in response to increased awareness of mental health needs among the target populations, as well as interest from funders.

The classical setup of a mental health relief deployment consists of a senior management team of expatriate mental health professionals, who on a rotational basis lead a multidisciplinary group of local staff on what is called a ‘mission's basis’. On the one hand, this collaboration is an excellent opportunity for capacity-building through transmission of skills and knowledge. However, NGOs’ work tends to focus largely on the short-term relief effort, even when the programmes remain for years, which translates into a limited margin of manoeuvre in adapting to evolving needs on the ground. Besides, these programmes often focus on common mental health disorders, providing psychosocial support but leaving individuals with severe mental disorders and substance misuse with limited access to services, especially in areas where government support is already scarce. Providing meaningful services to people in these two categories requires specialist mental health clinicians, primary care doctors with World Health Organization Mental Health Gap Action Programme training, accessible referral pathways and affordable medication protocols. It is a challenging setup that can quickly overwhelm budgets and operational capacity.

Although the interventions of NGOs in providing mental healthcare side by side with medical services can only be commended, a number of issues need to be highlighted in order to fill the gap in terms of mental health service delivery. It should be clear that mental health services’ timelines and goals are not similar to those of medical programmes. One particular concern is the fate of these programmes when conflicts go ‘cold’. Withdrawal of a previously non-existent service may destabilise a community that would have incorporated it into its resilience strategy.

## Recommendations

• A review of the available evidence and experience prompts us to compile a series of recommendations to ensure that populations in the Arab world receive mental healthcare that is responsive to conflict situations.
• The development of culturally relevant tools for assessment of the influence of conflict on pre-existing and emerging psychiatric conditions. At the least, a methodological adaptation of internationally validated tools should be undertaken, preferably at a pan-Arab level.
• Providing relevant training. A number of countries in the region have a long tradition of training the majority of their psychiatrists locally. This is particularly the case for Egypt, Saudi Arabia and the Maghreb. Newer training programmes have been created or grown in other member countries of the Gulf Cooperation Council (GCC) and in Lebanon more recently. This is an opportunity to expose the next generation of psychiatrists to best practice in the field of conflict-related mental healthcare. The same strategy should apply to psychologists, social workers and nurses.
• Better collaboration between local authorities and international organisations should begin from the onset of a crisis and involve the development of interrelated systems of care that can be sustained following the withdrawal of international aid, which is invariably the endpoint of any intervention. Overreliance on expatriate staff at a senior level is a major hurdle to the success of this step. Capacity-building at senior and middle management levels should be an early priority rather than an afterthought. An even better scenario would be to build capacity in low-and-middle-income countries deemed at risk even before they reach the situation of conflict.
• Research in the field of conflict psychiatric care is notoriously difficult to undertake for logistical and ethical reasons. This has meant that for decades interventions have been inconsistent and offered on the basis that ‘not doing anything’ was not an option. Understanding the limitations of established classification systems and traditional care models needs to be accompanied by researching alternative paradigms. An ethical framework is required that brings together academic institutions with relief organisations on the ground.^16^
• Finally, a joint effort by national psychiatric associations and opinion leaders in the Arab countries is needed in order to develop guidelines for assessment, diagnosis, treatment and management that are culturally sensitive and locally implementable. This can be under the auspices of the Arab Psychiatric Association or an equivalent structure and draw on successful experiences, including reliance on novel techniques such as telemental health.^17^

## Conclusions

The Arab world is currently going through a period of upheaval and violent conflict. Although some progress has been made over the past decades in mental health awareness and care, most unstable countries have underdeveloped and under-resourced mental health services. Negligible budgets are spent on large psychiatric hospitals, with limited investment in dynamic community mental health networks. A shortage of medication and other biological treatments, or adequately trained and supervised psychotherapists means that conflict-related psychiatric care is relegated to a luxury status and outsourced to internationally funded organisations. Better outcomes can be achieved in the short and long term when the unavoidable situation of conflict is converted into an opportunity for research, training and the building of local systems of resilience.

## Supplementary material

For supplementary material accompanying this paper visit https://doi.org/10.1192/bji.2020.41. click here to view supplementary material
